# Supplementary figures and images for: Mouse visual cortex as a limited resource system that self-learns an ecologically-general representation
Source: PLoS Comput Biol. 2023 Oct 2;19(10):e1011506. doi: 10.1371/journal.pcbi.1011506 (PMC10569538; doi:10.1371/journal.pcbi.1011506)

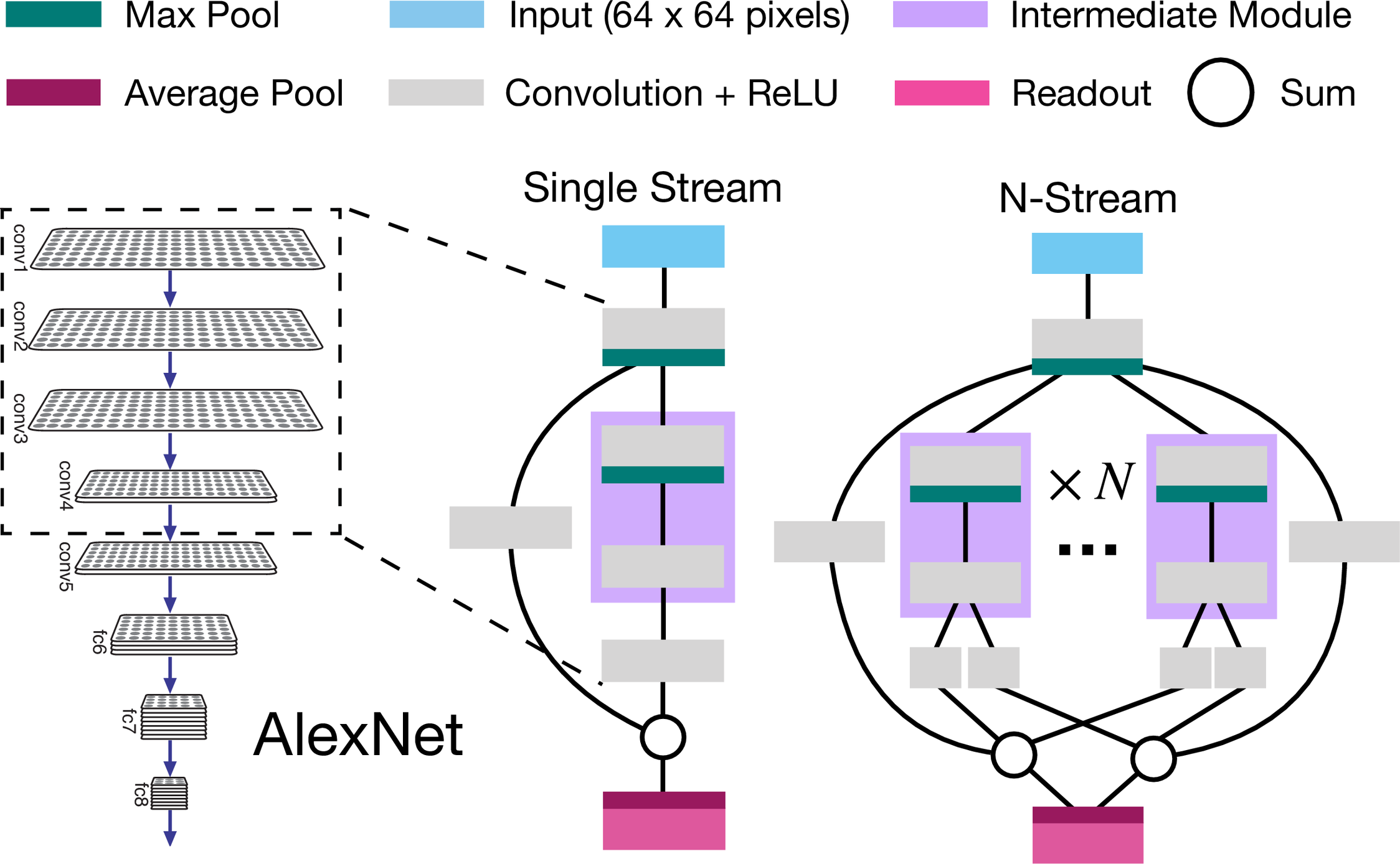

Supplement: S1 Fig — The first four convolutional layers of AlexNet best corresponded to all the mouse visual areas) These convolutional layers were used as the basis for our StreamNet architecture variants. The number of parallel streams, N, was varied to be one (single-stream), two (dual-stream) or six (six-stream). (TIF) [file pcbi.1011506.s001.tif]

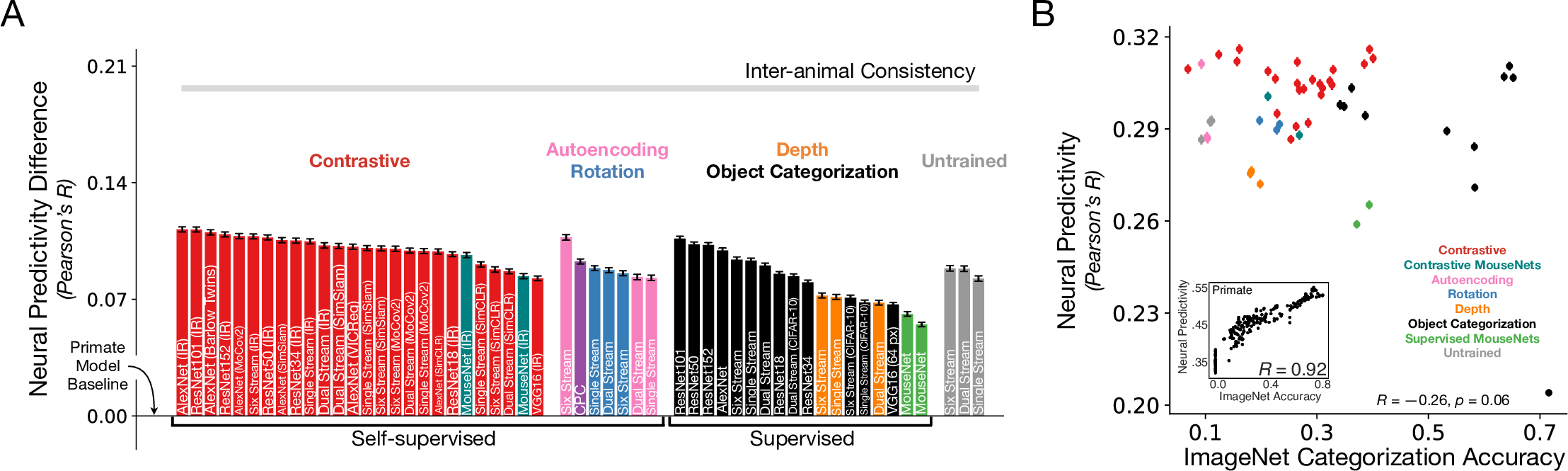

Supplement: S2 Fig — As in Fig 2, but for the calcium imaging dataset. A. The median and s.e.m. neural predictivity, using PLS regression, across neurons in all mouse visual areas except RL. N = 16228 units in total (RL is excluded, as mentioned in the “Neural Response Datasets” section). Actual neural predictivity performance can be found in Table 2. The “Primate Model Baseline” denotes a supervised VGG16 trained on 224 px inputs, used in prior work [14, 15, 36]. All models, except for the “Primate Model Baseline”, are trained on 64 px inputs. B. Each model’s performance on ImageNet is plotted against its median neural predictivity across all units from each visual area. Inset. Primate ventral visual stream neural predictivity from BrainScore is correlated with ImageNet categorization accuracy (adapted from Schrimpf et al. [17]). All ImageNet performance numbers can be found in Table 2. Color scheme as in A and Fig 2A. (TIF) [file pcbi.1011506.s002.tif]

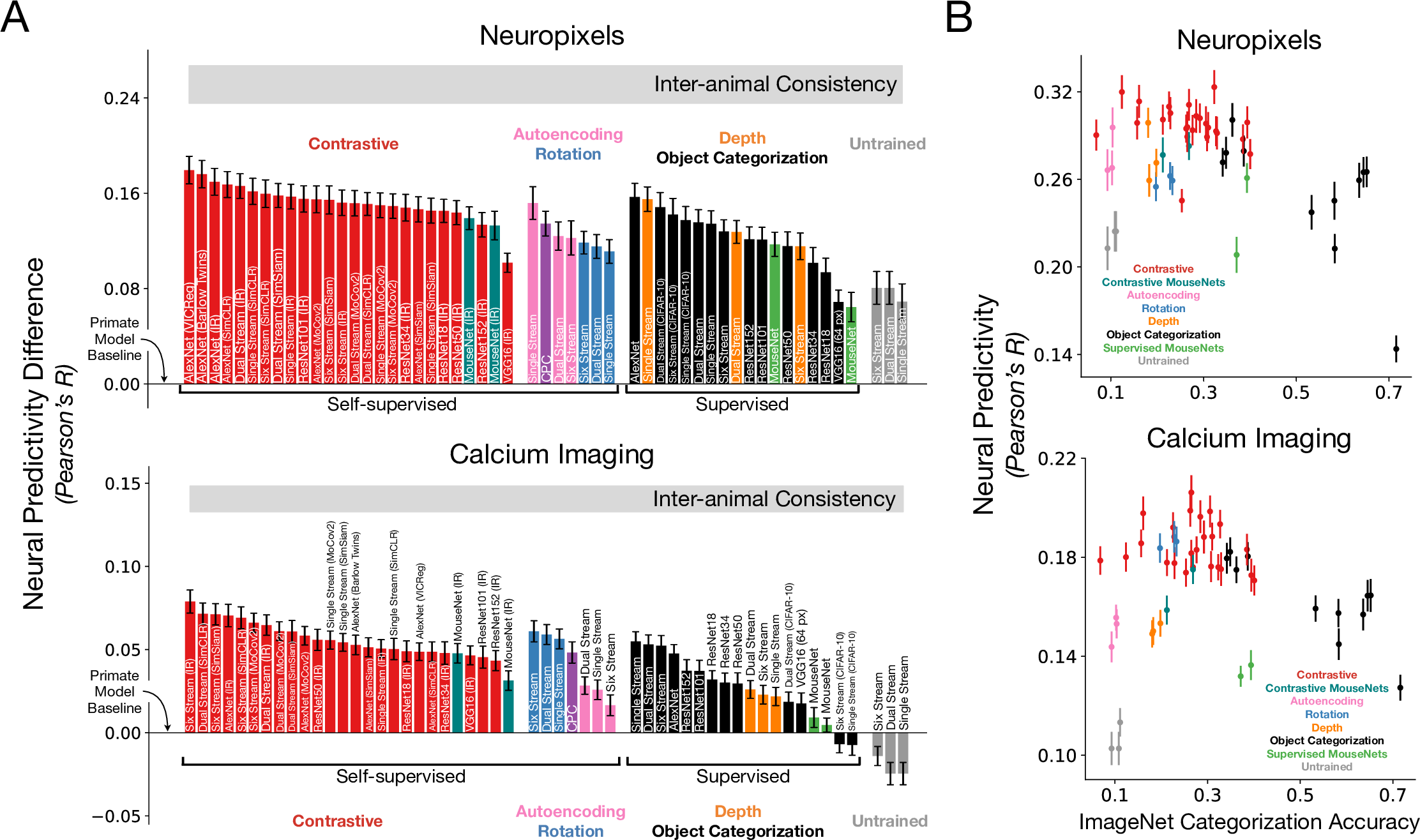

Supplement: S3 Fig — A. The median and s.e.m. noise-corrected neural predictivity, using RSA, across N = 39 and N = 90 animals for the Neuropixels and calcium imaging dataset respectively (across all visual areas, with RL excluded for the calcium imaging dataset, as mentioned in the “Neural Response Datasets” section). The “Primate Model Baseline” denotes a supervised VGG16 trained on 224 px inputs, used in prior work [14, 15, 36]). All models, except for the “Primate Model Baseline”, are trained on 64 px inputs. B. We plot each model’s performance on ImageNet against its median neural predictivity, using RSA, across visual areas. All ImageNet performance numbers can be found in Table 2. Color scheme as in A and Fig 2A. (TIF) [file pcbi.1011506.s003.tif]

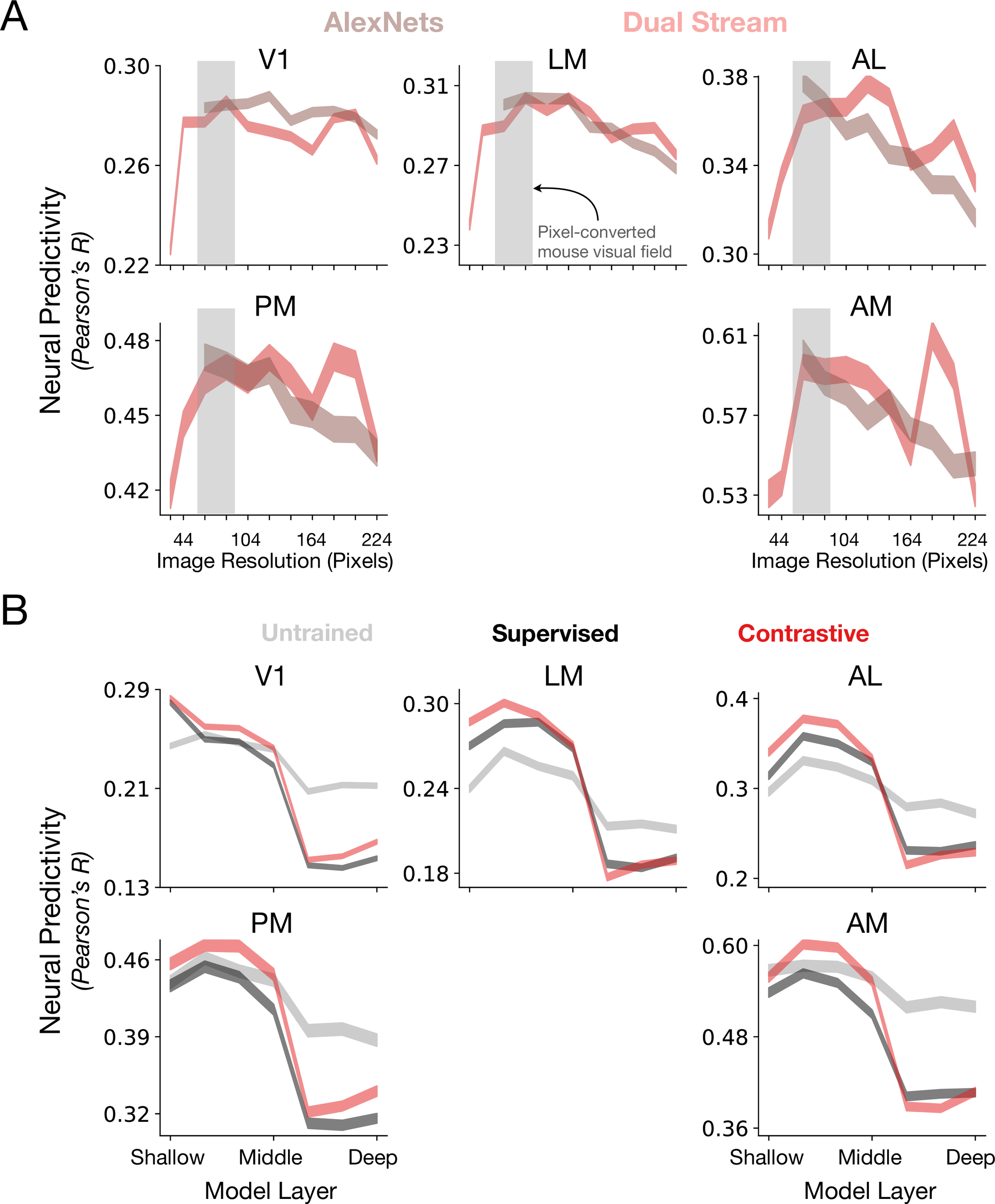

Supplement: S4 Fig — A. As in Fig 3, our dual stream variant (red) and Contrastive AlexNet (brown) were trained using lower resolution ImageNet images and in a contrastive manner. Each image was downsampled from 224 × 224 pixels, the image size typically used to train primate ventral stream models, to various image sizes. Training models on resolutions lower than 224 × 224 pixels generally led to improved neural predictivity. The median and s.e.m. across neurons in each visual area is reported. As mentioned in the “Neural Response Datasets” section, visual area RL was removed from the calcium imaging neural predictivity results. Refer to Table 1 for N units per visual area. B. As in Fig 2B, AlexNet was either untrained, trained in a supervised manner (ImageNet) or trained in an self-supervised manner (instance recognition). We observe that the first four convolutional layers provide the best fits to the neural responses for all the visual areas while the latter three layers are not very predictive for any visual area. As mentioned in the “Neural Response Datasets” section, visual area RL was removed from the calcium imaging neural predictivity results. (TIF) [file pcbi.1011506.s004.tif]

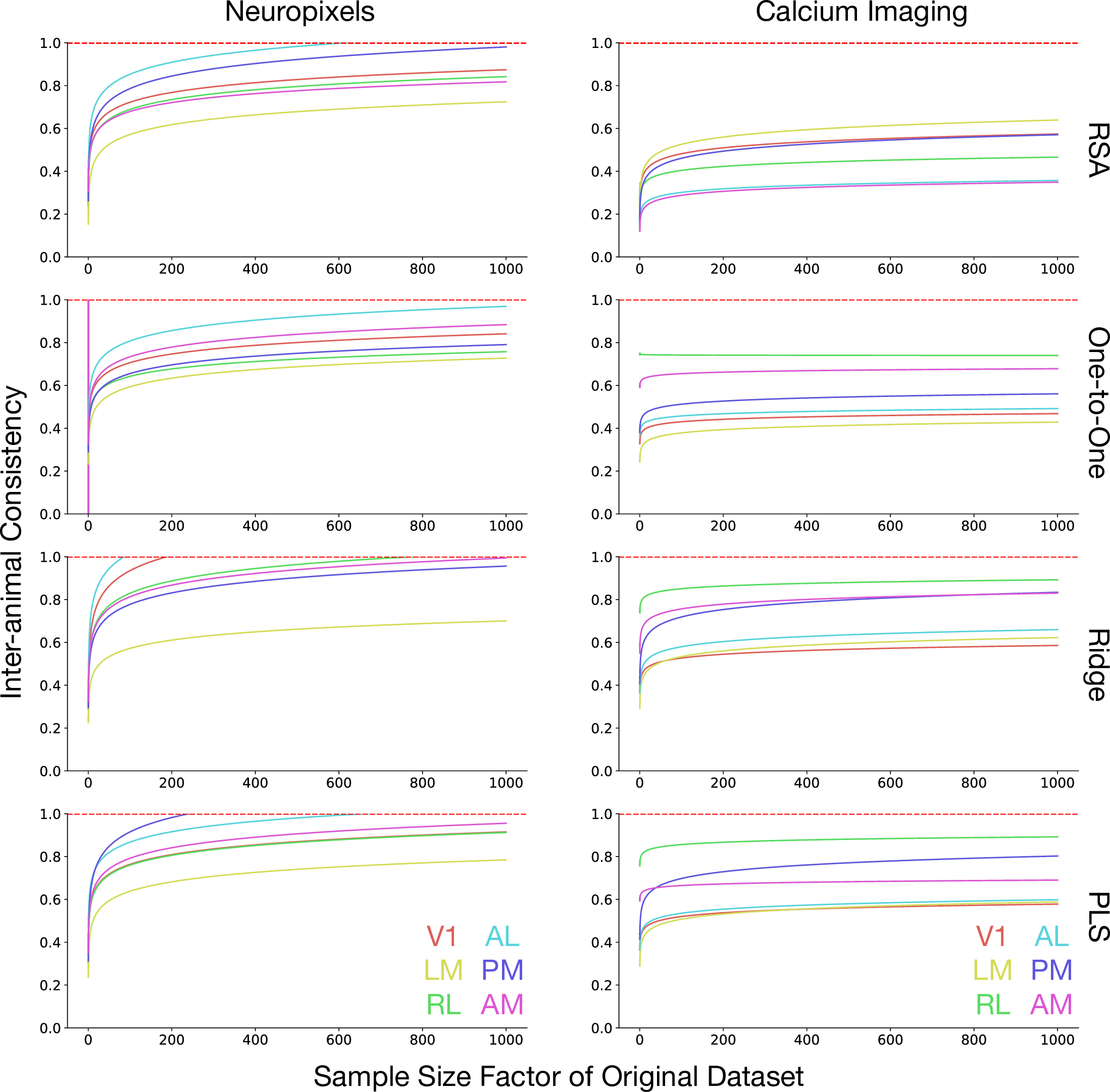

Supplement: S5 Fig — Inter-animal consistency was extrapolated across the number of units in each dataset using a log-linear function (f(n) = a log10(n) + b, where a and b are fit as parameters via least squares, and n is the sample size factor). This analysis reveals that inter-animal consistency of the Neuropixels dataset approaches 1.0 more rapidly than it does for the calcium imaging dataset. Inter-animal consistency evaluated at a sample size factor of one indicates the consistency when all the existing units in the datasets are used (i.e., inter-animal consistency values reported in Fig 1A). (TIF) [file pcbi.1011506.s005.tif]

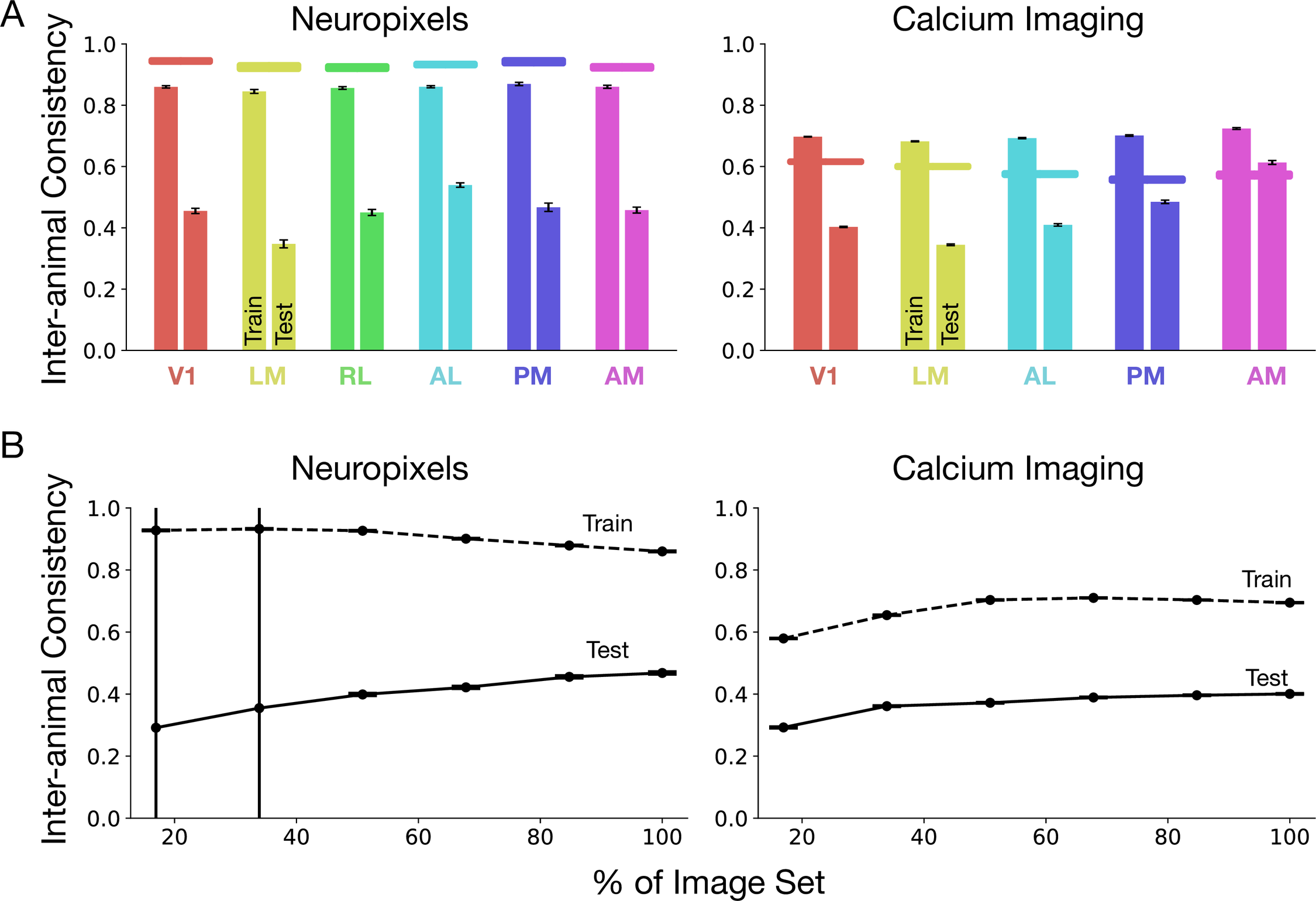

Supplement: S6 Fig — A. Inter-animal consistency under PLS regression evaluated on the train set (left bars for each visual area) and test set (right bars for each visual area), for both Neuropixels and calcium imaging datasets. The horizontal lines are the internal consistency (split-half reliability). B. Inter-animal consistency under PLS regression on the train set (dotted lines) and test set (straight lines), aggregated across visual areas. Each dot corresponds to the inter-animal consistency evaluated across 10 train-test splits, where each split is a sample of the natural scene image set corresponding to the percentage (x-axis). Note that RL is excluded for calcium imaging, as explained in the text (the “Neural Response Datasets” section). The median and s.e.m. across neurons is reported for both panels. Refer to Table 1 for N units per visual area. (TIF) [file pcbi.1011506.s006.tif]

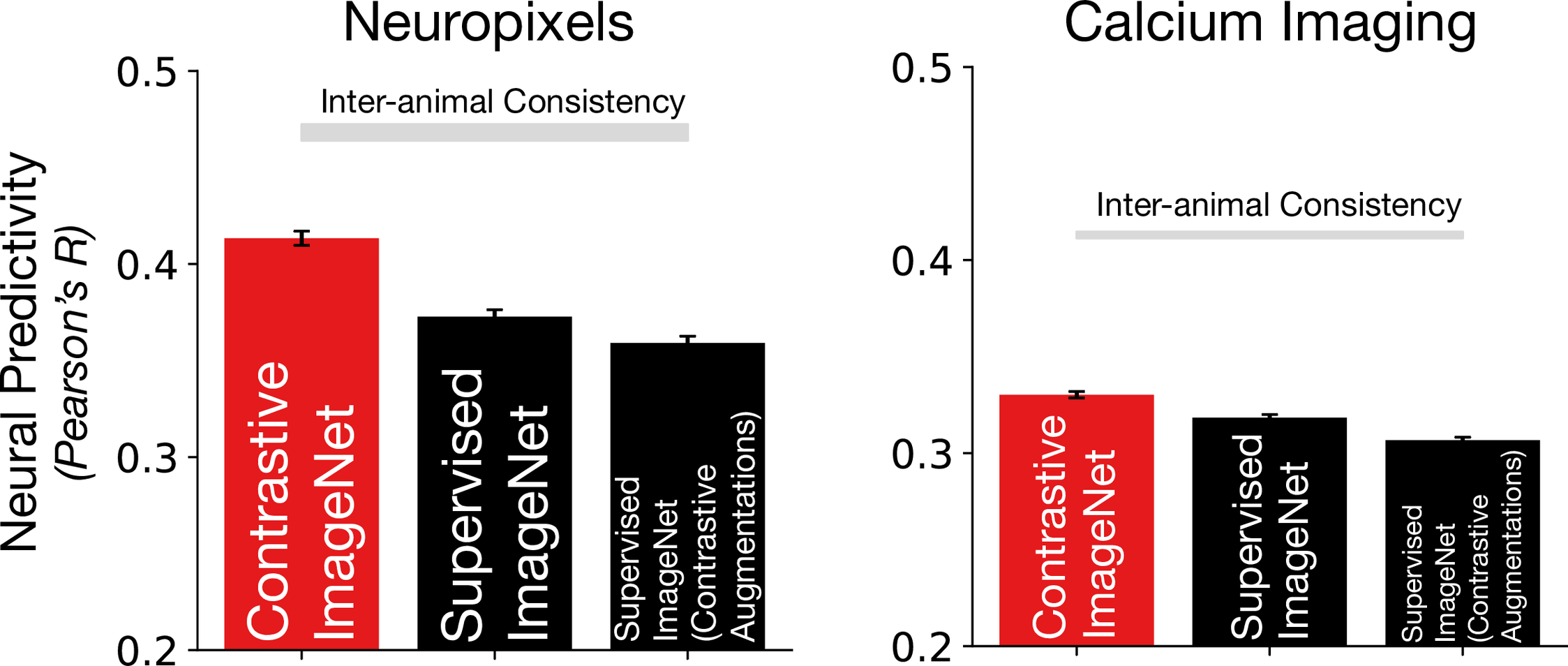

Supplement: S7 Fig — Here we compare the neural predictivity of AlexNet trained in three different ways. Contrastive ImageNet is an AlexNet trained using instance recognition on ImageNet with augmentations that are part of the contrastive algorithm (random crop, random color jitter, random grayscale, random horizontal flip). Supervised ImageNet is an AlexNet trained on ImageNet in a supervised manner with a smaller set of augmentations (random crop and random horizontal flip). Supervised ImageNet (contrastive augmentations) is an AlexNet trained on ImageNet in a supervised manner with the augmentations used in the instance recognition algorithm. This control model allows us to ascertain whether the improved neural predictivity of the Contrastive ImageNet model (red) is due to the contrastive loss function itself or due to the larger set of image augmentations used during model training. In both neural response datasets, we can conclude that data augmentations alone do not contribute to improved correspondence with the mouse visual areas. (TIF) [file pcbi.1011506.s007.tif]

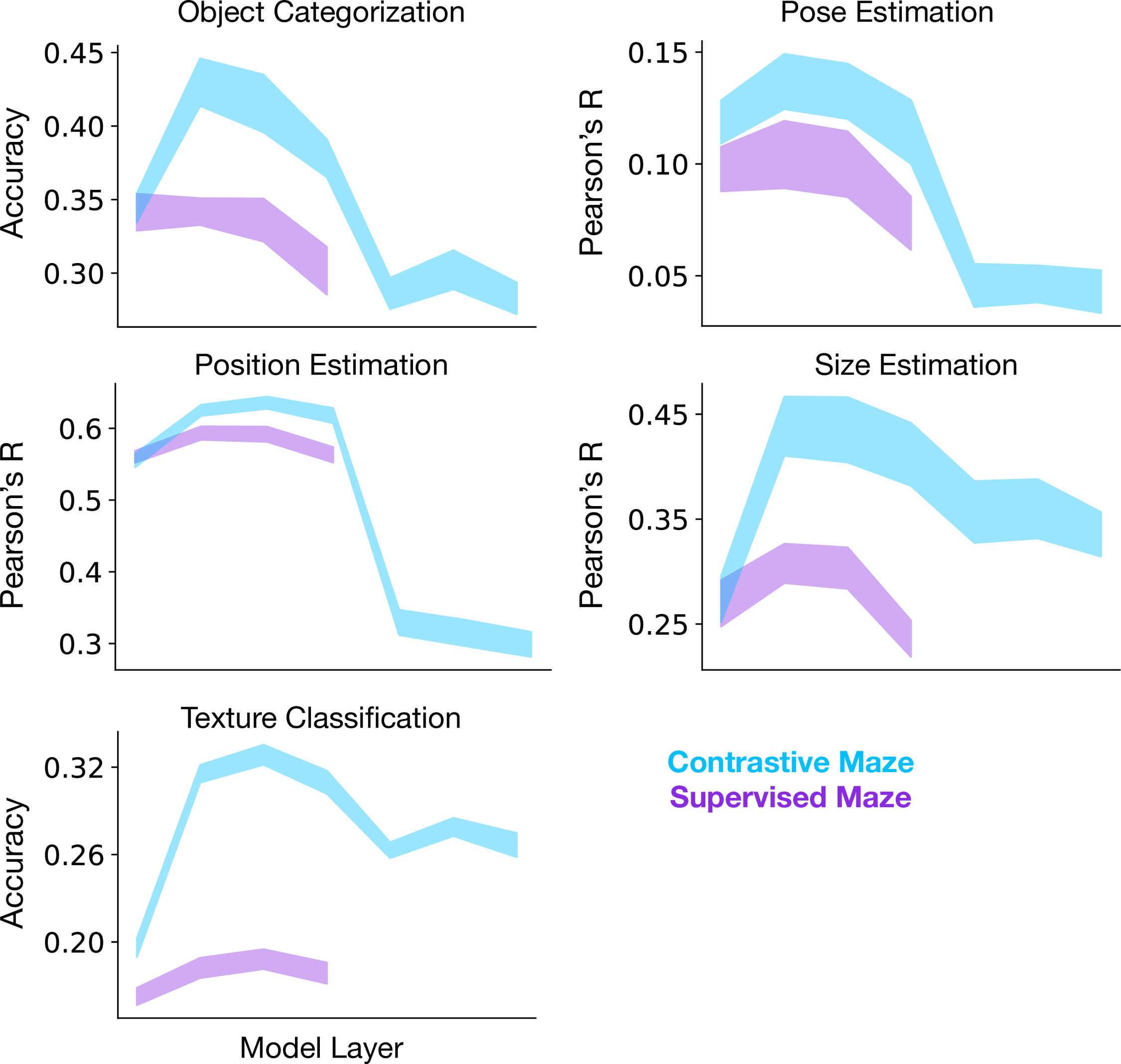

Supplement: S8 Fig — For the models trained on images from the maze environment, we plot their transfer performance on a set of out-of-distribution tasks (described in lower right panel of Fig 6C) across model layers. We find that intermediate model areas are better able to perform the transfer tasks and that the model layers that attain peak performance on the tasks correspond to those that best predict neural responses in the intermediate/higher mouse visual areas (see Fig 2B). (TIF) [file pcbi.1011506.s008.tif]

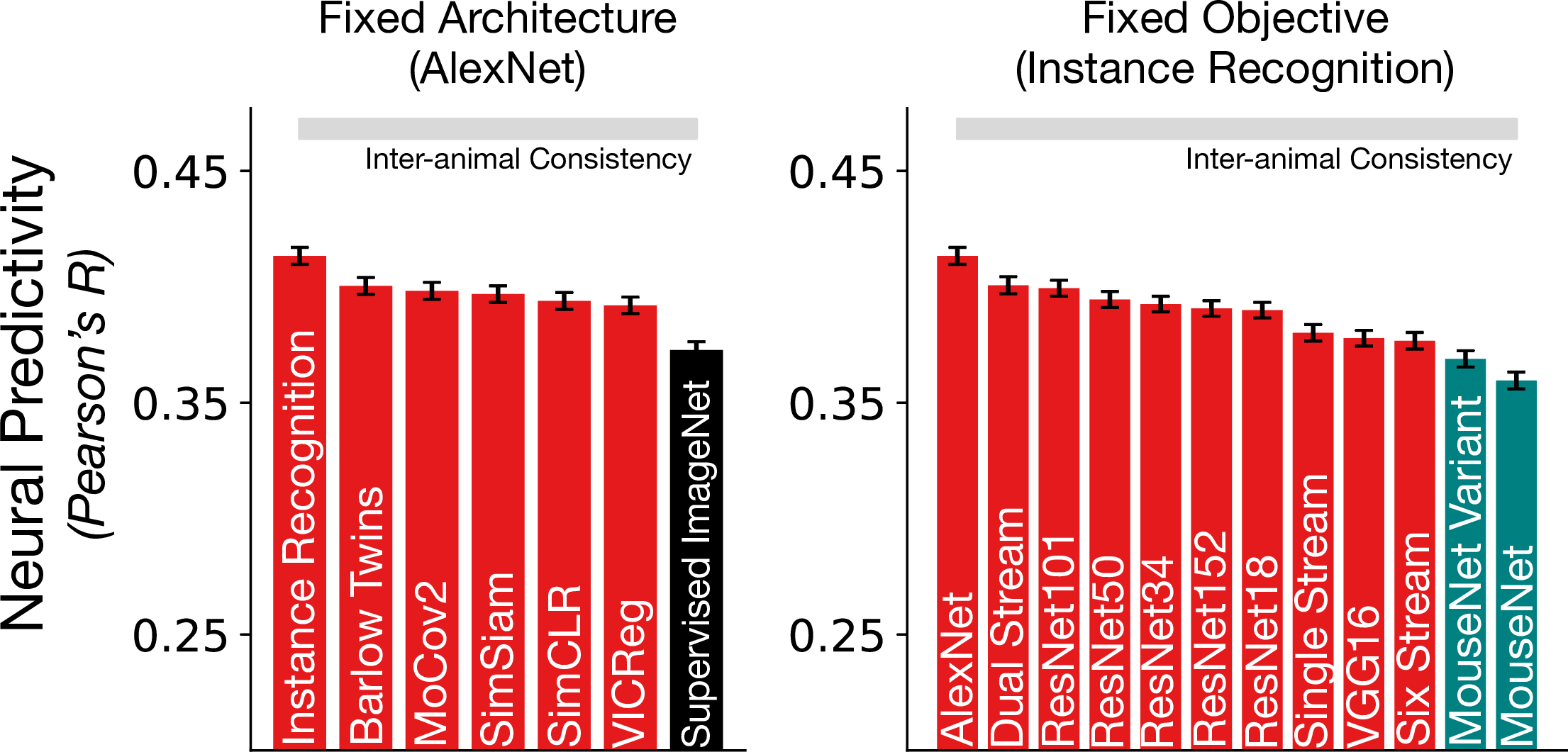

Supplement: S9 Fig — The data in the left panel shows how neural predictivity varies when the architecture is fixed to be AlexNet, but the objective function is varied between supervised (object categorization) and self-supervised, contrastive objectives. The data in the right panel shows how neural predictivity varies when the objective function is fixed to be instance recognition, but the architecture is varied, including StreamNets, MouseNets, VGG16, ResNets, and AlexNet. (TIF) [file pcbi.1011506.s009.tif]

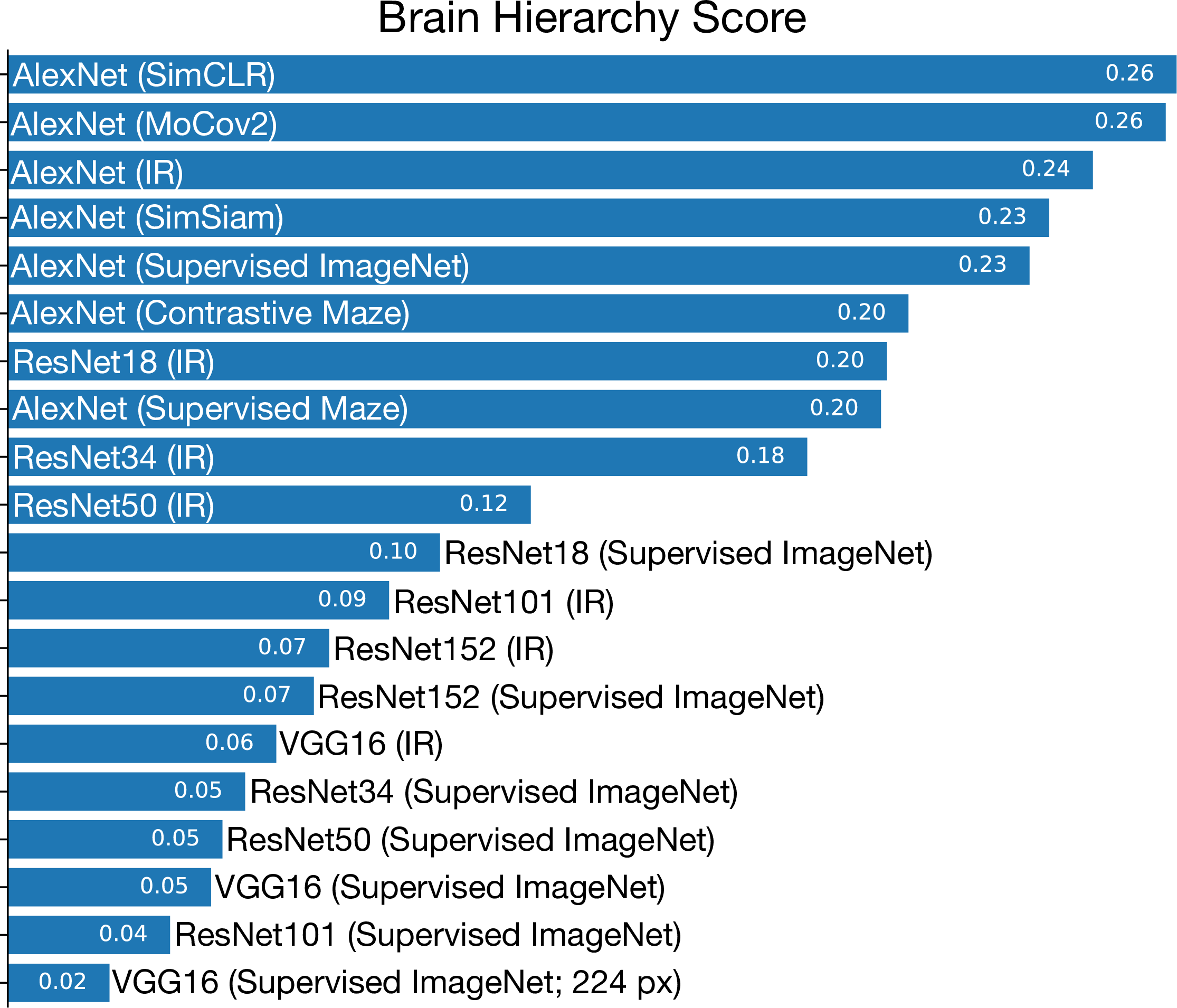

Supplement: S10 Fig — The brain hierarchy score metric of Nonaka et al. [51] was computed for a set of feedforward CNNs. Self-supervised, contrastive models (and shallower models) have a higher brain hierarchy score, computed using the mapping from model features to electrophysiological responses. (TIF) [file pcbi.1011506.s010.tif]
