# Supplementary material for: Mouse visual cortex as a limited resource system that self-learns an ecologically-general representation
Source: PLoS Comput Biol. 2023 Oct 2;19(10):e1011506. doi: 10.1371/journal.pcbi.1011506 (PMC10569538; doi:10.1371/journal.pcbi.1011506)
Supplement: Attachment — Submitted filename: Mouse_Vision_PLoS_Response.pdf [file pcbi.1011506.s011.pdf]

We thank the reviewers for their thoughtful comments and thorough evaluation of our manuscript. We have significantly revised the paper to address the main concerns of the reviewers, including additional experiments to further strengthen our claims. We really appreciate their time and effort, as their suggestions have improved the clarity of the manuscript, and as a result, we have acknowledged them in the revised manuscript.

Additionally, at the time of initial submission, per the journal guidelines (https://journals.plos.org/ploscompbiol/s/code-availability), we had shared a private version of our repository (https://github.com/neuroailab/mouse-vision) to the PLoS Comp editorial board email address (ploscompbiol@plos.org) to share with reviewers, but it does not seem that this repository was forwarded to the reviewers. We will make this same repository link public upon acceptance, and have included this link in the "Code Availability" section of the revised manuscript. In the meantime, we have also uploaded it as a zip file under "Supporting Information -- Compressed/ZIP File Archive".

We summarize the largest changes below:

1. **A new Section 2 and Figure 1 in the main text, right before the results section (now Section 3), that motivates and describes the inter-animal consistency analysis and neural datasets used.**
2. **Expanded descriptions of the various models used in Section 3 and clear references to the bars they represent in Figure 2A, along with an expanded Figure 2 caption explaining these points as well.**
3. **Four new models and their comparisons to data (updated in Figures 2, S2, and S3): Contrastive versions of the Shi MouseNets, and AlexNet trained with VICReg and Barlow Twins. Explanation of contrastive definition in Section 3.2 (on objective functions) pp. 7-8. Our original model, Contrastive AlexNet (renamed from Contrastive ShallowNet based on reviewer comments), remains the best predictor of the data.**
4. **Additional supplemental Figures S8, S9, and Table S4 that answer reviewer questions about out of distribution task performance across model layers and neural predictivity with architecture and objective function fixed.**

Point-by-point responses:

Reviewer #1

Nayabi, Kong et al. compared the performance of models trained with supervised and self-supervised learning in predicting the responses of mouse visual areas to natural stimuli. Their results showed an outperformance of Shallow architectures trained with SSL compared to other models. In addition, the SSL-trained models showed better out-of-distribution generalization, based on which the authors suggest that the mouse visual cortex can be understood as a shallow general purpose visual system. I enjoyed reading this paper, and believe it offers a strong contribution to the comparison of visual systems across different species using deep neural networks. I have a few concerns that I elaborate below.

Major comments:

1) If the focus of the paper is on evaluating the importance of contrastive methods for predicting the responses of mouse visual areas, it would be important to include comparisons with non-contrastive SSL techniques, such as Barlow Twins, VICReg, and others. Alternatively, if such comparisons are not included, it may be more appropriate to use a different terminology rather than 'contrastive' for SSL models.
Thank you for mentioning this point! We find that "contrastive" is a helpful grouping term since unlike prior SSL methods, all of these methods learn embeddings that are at least invariant/robust to augmentations, and therefore have to contrast against *something* to avoid representation collapse – even if they aren't explicitly negative batch *examples*. One such method we included in our initial submission, that doesn't use negative batch examples, is SimSiam, which relies on asymmetric representations via a stop gradient to avoid collapse. Barlow Twins relies on regularizing with the cross correlation matrix's off diagonal elements, and VICReg uses the variance and covariance of each embedding to ensure samples in the batch are different. We clarify this in paragraph 5 of Section 3.2, when we first introduce these objective functions.

As an additional analysis, we trained AlexNet with both the Barlow Twins and the VICReg objectives, and observed comparable neural predictivity (Figure 2A and Table S3) with the remaining Contrastive SSL methods in the red bars (including SimSiam). We include these results in the revised version of the manuscript, and we thank the reviewer for this important clarification.

2) Regarding Section 2.1, I found it unclear why the mapping method that resulted in the highest inter-animal similarity (PLS) was chosen as the similarity measure, especially considering that the correlation values across different metrics are not that different. It would be helpful to understand the range of these differences and why a higher value is considered indicative of true similarity. Additionally, it would be interesting to know how the results might differ if a different metric, such as CKA, were used instead. It is important to explicitly explain the differences in results obtained with different similarity measures in the main text.
We chose PLS regression both to compare directly to prior primate ventral stream results (which use this mapping) to better understand ecological differences between the two visual systems across species, and to identify the mapping that enables the animals to be most similar to each other. The latter property is preferred since we judge models to be at least as similar as any animal is to another animal (in other words, the model is an "in-silico" stand-in animal). Therefore, we want to ensure we are studying the inter-animal similarity with high fidelity, to be in a position to best separate models. In other words, if the inter-animal consistency is too low under a given transform class, then our models may seem like they are doing better at explaining the neural response patterns. We expand on this motivation for the analysis entirely in its own section (Section 2), preceding the results section (now Section 3 instead of Section 2, in the

revised manuscript). We also include RSA results in the Appendix (Figure S3), and observe similar trends as with PLS regression which we note in Section 3.1.

3) The authors employed the Contrastive ShallowNet architecture, which comprises the first four layers of AlexNet trained with contrastive SSL. I wonder why the authors did not solely use a 4-layer convolutional ANN and train it with contrastive SSL if a shallow architecture is sufficient for the purpose of explaining the responses of mouse visual areas.
We did try using a four-layer convolutional ANN and trained them with contrastive SSL. Specifically, we developed the StreamNets, which consist of four convolutional layers with a skip connection from the first convolutional layer to the last convolutional layer (see Figure S1 for its schematic). We found that such StreamNets resulted in high-performing models of mouse visual cortex, but were not as good as Contrastive ShallowNet (which we refer to as "Contrastive AlexNet" in the revised manuscript, for greater clarity).

4) I find the argument presented in Figure 3 for the role of depth in predicting mouse visual cortex responses unconvincing, as there are several confounding factors that make the interpretation difficult. Eg, some models listed in fig 3 are based on AlexNet architecture, while others use ResNet architecture (also some architectures other than AlexNet are listed in fig 1 despite the claim that only AlexNet was used in the experiments). Additionally, some models were trained with contrastive objectives, while others were trained with supervised objectives. Even within the contrastive methods, there are different contrastive objectives. It would be more compelling if the effect of architecture depth was demonstrated by controlling for other factors, such as the objective function.
We apologize for the lack of clarity here. We therefore added a new supplemental figure (Figure S9) isolating such factors. In the right panel of Figure S9, we see that when the objective function is fixed to be instance recognition, models with a fewer number of convolutional layers (in sequence) best predict the mouse visual responses. Specifically, models with only four convolutional layers (StreamNets; Single, Dual, or Six Streams) perform as well as or better than models with many more convolutional layers (e.g., compare Dual Stream with ResNet101, ResNet152, VGG16, or MouseNets). We now refer to this figure in Section 3.4.

5) The reward-based navigation task presented in fig 6A requires further explanation. What is the goal of the task? What kind of visual features are informative in solving the task effectively? It is hard to conceive how a model trained on static images would be useful for a dynamic behavior like navigation. Moreover, to better understand the differences between SSL and supervised models in relation to this task, it would be helpful to compare them with a model directly trained with RL on the task in terms of the mean episode return.
The goal of the task can be seen in Figure 6C upper right, where the goal of the agent is to collect rewards in the form of "blue orbs". You are correct in that the models (i.e., visual backbones) were trained on static images and therefore are able to extract "good" visual representations (in the sense that they are useful for transfer tasks such as ImageNet categorization). However, when training the agent with the visual backbone to

perform the reward-based navigation task, the visual features of each frame in the episode are integrated using an LSTM (shown in Figure 6B, old Figure 5B), so that the *history* of the visual features in each episode can (in principle) be used by the agent to inform future actions. When we directly train a model using RL on the reward-based navigation task (i.e., we do not pretrain the visual backbone on ImageNet images), we see that it is able to obtain greater episode return, as expected. However, it is insufficient to improve neural predictivity on the visual responses, shown by the purple bar in Figure 7C (previous Figure 6C).

We also added a table in Section 6.7 (Table S4) showing the mean episode return for RL agents that use each of the visual backbones. As expected, models that were trained using the visual statistics of the maze environment obtain more rewards than those trained on ImageNet.

6) I found the purpose of the experiments with the virtual rat unclear. If similar experiments were conducted using a virtual monkey simulation, would we expect to observe similar results? How would the interpretations differ? Is there anything specific in the rat's movement repertoire or visual experience that necessitates the use of representations learned with SSL?
The primary purpose of the experiments with the virtual rodent weren't to necessarily make a specific statement about rat *movements*, but mainly to test the setting of using an SSL optimized visual encoder (vs. its supervised counterpart) to control a high-dimensional animal body with high-dimensional continuous inputs – a problem that many (if not all) animals have to solve. Given that we were trying to better understand why SSL methods be better predicting mouse visual cortical neurons, we did try to stick to a reasonable ecological task for that species (e.g. navigation) and that its affordances were somewhat similar to that of an actual rodent via the biomechanical realism of its body. In particular, if we used our shallow, lower acuity SSL encoder for controlling a virtual monkey simulation for a task that a monkey is adapted to (e.g., object manipulation) that this would not work that well given that this likely requires high visual acuity and good object recognition abilities at a minimum. We clarify this motivation in paragraph 3 of Section 4 of the revised manuscript, and thank the reviewer for bringing this point up.

7) I noticed that the results of the Neuropixel and calcium imaging data are not entirely consistent. Specifically, the differences between the performance of the best SSL and supervised models versus autoencoding models do not appear to be significant with calcium imaging based on fig S3. Could the authors provide any explanation for these discrepancies? I am curious whether the deconvolved spike timings or calcium traces were used in these experiments.
The calcium imaging responses were obtained from the average of the df/F trace (see here). We additionally find that the calcium imaging data is less reliable than the Neuropixels data, both with PLS regression and RSA (namely, animals are less reliably consistent with one another, as shown by the inter-animal consistency being lower in the gray horizontal lines of Figures S2 and S3; and even as a function of subsampled units

across all areas and transforms in Figure S5), which is why we rely on the newer and more reliable Neuropixels dataset for the main results in our work, as it is in a better position to differentiate models. Additionally, recent independent analysis from the Allen Institute (Siegle et al., 2020), found that the Neuropixels dataset does not suffer from issues of field-of-view localization as the calcium imaging dataset did. We extensively mention these points in a new Section 2 of the revised manuscript, prior to the main results section. We thank the reviewer for bringing this point up, which helped us clarify it in the revision.

8) Could the intrinsic dimensionality of the learned representations account for the transferability of the SSL model?

Thanks for this interesting question. We performed this new analysis for each of the four models shown in Figure 7 (Contrastive ImageNet, Supervised ImageNet, Contrastive Maze, Supervised Maze; old Figure 6). For each mode, we computed the effective dimensionality (ED) of each model layer, where effective dimensionality is quantified as follows:

$$\mathrm{ED} = \frac{\left(\sum_{i=1}^{K} \lambda_i\right)^2}{\sum_{i=1}^{K} \lambda_i^2}$$

In the equation above, λ_i, denotes the *i*-th eigenvalue of the covariance matrix of the model responses at each layer and *K* denotes the total number of eigenvalues for the model responses. Shown in the figure below, we find that the contrastive models (in orange) have higher effective dimensionality (across most of the model layers) than their supervised counterparts (in blue). Note that the Supervised Maze model is a visual backbone constructed from the first four layers of AlexNet.

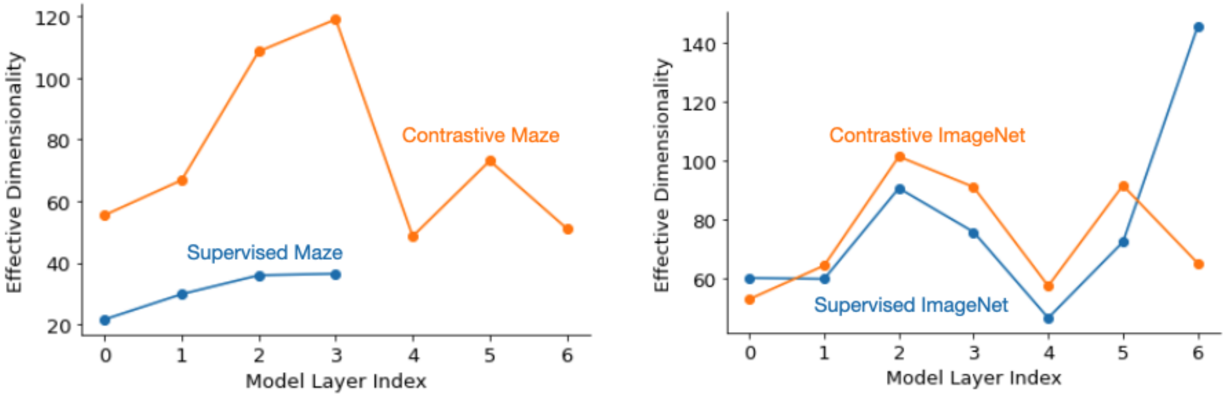

Minor comments:

1) What are error bars when showing models' predictivity difference e.g. fig 1A)?I suggest that the authors provide more clarity in reporting the statistical differences between the various models.

The error bars in Figure 2A (previous Figure 1A) show the standard error of the neural predictivity across units. It is now more explicitly stated in the caption.

2) The Color scheme needs to be added to fig 3.
The color scheme is now added to Figure 4 (previous Figure 3).

3) The details of the mouse visual cortex data, stimuli etc needs to be included in the results.
Thank you for this suggestion. We have included the details of the visual cortex data and stimuli in paragraph 3 of the new Section 2, which introduces the inter-animal consistency analysis and the datasets for the first time.

Reviewer #2

This work identifies several important features that make a vision model a better fit for mouse versus primate vision. These features reflect known differences between the two systems such as architecture depth and image resolution. My concerns are mainly around clarity and presentation, with some requests for extra analyses.

Clarity:

Given that aspects of interanimal consistency as a metric are discussed throughout, I think it would make sense to include fig S1 or some variant of it in the main text in order to clarify the metrics and to motivate the focus on neuropixels over calcium imaging.
We thank the reviewer for this suggestion to improve the clarity of the manuscript. We greatly expand on this motivation for the analysis in Section 2.1 as its own earlier Section 2 (prior to the main results, which is now in Section 3), and move previous Figure S1 to the main text as the new Figure 1.

I found the choice to refer to supervised VGG-16 as the "prior primate model" confusing, as many different models have been used to predict neural activity in the primate and giving it this title suggested to me it was somehow more fit to primate data than a standard VGG16. I'm fine with the authors explaining this choice as the baseline due to its common use as a primate model, but I think in the figures and discussion of results it should just be labeled as supervised VGG-16.
We thank the reviewer for this suggestion, and we have clarified why we label VGG16 as the "Primate Model Baseline" in the first paragraph of Section 3.2 (on pg. 5 including footnote 3), given that it was the de facto CNN used in the initial goal-driven modeling studies of mouse visual cortex (Cadena et al., 2019b; Shi et al., 2019; DeVries et al., 2020), and our aim is to illustrate how the three factors that we study, which deviate in visual acuity, cortical depth, and function objective from the primate ventral stream, quantitatively improve these models greatly. We also explicitly define that VGG16 corresponds to the "Primate Model Baseline" in the caption of Figure 2 (previously Figure 1) in the revision.

In section 2.2, there was not an explicit enough distinction between image set and objective. For example, ImageNet could be considered "human-centric" by the nature of the images themselves, not just the fact that category labels are applied to them, and this aspect of the data would impact both supervised and self-supervised methods. The idea of using fewer categories is also conflated with the type of images (CIFAR-10 is not just fewer categories, it is smaller and lower quality images as well). I would like the description of the models in this section to acknowledge when different image sets are used in addition to different training objectives.
Thanks for this great point and for your clarification. We have now mentioned, in Section 3.2 (where we describe all the models), the human-centric nature of the images themselves, and the lower-quality nature of CIFAR-10. We also made clear in the caption of Figure 2A (previous Figure 1A) and Section 3.2 (previous Section 2.2) that all models are trained using ImageNet, except for the CPC model (purple), depth prediction models

(orange), and the CIFAR-10 supervised models (CIFAR-10-labeled black bars). Also, where possible, individual comparisons are made, amongst the best architectures and loss functions for a *fixed* dataset, see the new supplemental Figure S9, which we reference in multiple sections in the main text, including Section 3.2.

I did not find Figure 3 very valuable. The depth of the overall network is not the same as the number of LNL operations that occur before the units responsible for the neural prediction (as 1B shows much of the predictive power is coming from early layers). It seems the latter should be used if the goal is to show that not many LNLs are needed to predict mouse visual responses.
We just wanted to show that even if the number of convolutional layers is minimal, when trained with the "right" objective (i.e., self-supervised, contrastive), the models can attain high neural predictivity, on par with models with many, many more convolutional layers (e.g., 4-layer models vs. 152-layer models).

Relatedly, I am confused as to why the authors choose to refer to their best-performing model as "Contrastive ShallowNet" versus simply Contrastive AlexNet. The architecture of the trained model is AlexNet. The fact that the first four layers are most predictive of mouse response is useful to know, but it does not make the model as it was built any shallower. I imagine many of the other networks also rely primarily on earlier layers for their predictions, but they are not referred to as a separate model as result of this. If the authors showed that a model trained with a shallower architecture was better than I would understand, but as it stands now this name change is confusing and is not applied evenly across all models.
Sorry for the confusion. We have clarified this and refer to the best-performing model as "Contrastive AlexNet". All the figures and captions have been updated to reflect this change.

In figure 6, why are not all models evaluated on all tasks? At the very least, it is important to include the reward performance of the 'supervised' maze model in Fig 6A in order to contextualize these numbers.
We did not evaluate all the models on all the tasks because: 1) we wanted to assess the task generalization capability of the *best* model of mouse visual cortex, namely, Contrastive AlexNet, and 2) what benefits in task generalization are conferred if the AlexNet architecture is trained in a contrastive manner versus in a supervised manner in either training environment. Therefore, because this figure is focused on the point of task generalization, we do not include the reward performance of the "supervised" maze model in Figure 7A (old Figure 6A) since this is trained in the same environment that it is being evaluated on, unlike the other models in that figure. For contextualization of these numbers, we include the mean episode return for all four models in a new Table S4 in Section 6.7, where the RL task is described.

Relatedly, I'd like to see the authors discuss how these results contrast with Lindsay et al, which shows somewhat better match to mouse data with the an RL-trained model over

supervised/unsupervised methods (knowing how well the RL trained visual system here works may be important for this comparison)

The self-supervised methods (of autoencoding variants and CPC) that Lindsay et al. use are known to be less powerful representation learners than the more current state of the art methods we used. We compared autoencoding and CPC in Figure 2 (previous Figure 1), and showed that these were worse than our best self-supervised methods at matching neural response patterns. This is therefore consistent with our original findings that training end-to-end with an RL loss, as Lindsay et al. did, performed worse at predicting the neurons than our best self-supervised models, shown by the purple bar in Figure 7C (previous Figure 6C). We have added an explanation of these points in the paragraph 8 of Section 4.

Additionally, our study goes beyond Lindsay et al. in several ways:

1. Their transfer tasks are not evaluated on out of distribution image sets. Furthermore, they do not embed the different supervised & unsupervised encoders into the RL agent to evaluate downstream task performance.

2. Their visual encoder architecture is fixed to be a ResNet, and they do not vary it like we do. Additionally, we study the effect of varying image resolution during model training on the ability to match mouse visual responses.

3. Their “supervised models” are never trained on image categorization at low resolution, or depth estimation – the latter would be a proxy for sensing 3D objects with a rodent’s whiskers.

4. They use only one metric (RSA), and do not also try linear regression (we use both).

5. They only compare to calcium imaging data, not additionally to the newer (and more reliable) Neuropixels dataset (we compare to both).

The authors state "as we find that higher model areas best support these scene understanding transfer tasks". Was this shown somewhere?

We apologize for omitting the data showing that higher model areas better support scene understanding tasks. We add a new supplemental figure (new Figure S8) that shows data supporting this claim and reference the figure in the last paragraph of Section 3. For the models trained on images from the maze environment, we plot their transfer performance on a set of out-of-distribution tasks (described in lower right panel of new Figure 6C [previous Figure 5C]) across model layers. In the figure shown below, we find that intermediate model areas are better able to perform the transfer tasks and that the model layers that attain peak performance on the tasks correspond to those that best predict neural responses in the intermediate/higher mouse visual areas (see new Figure 2B [previous Figure 1B]).

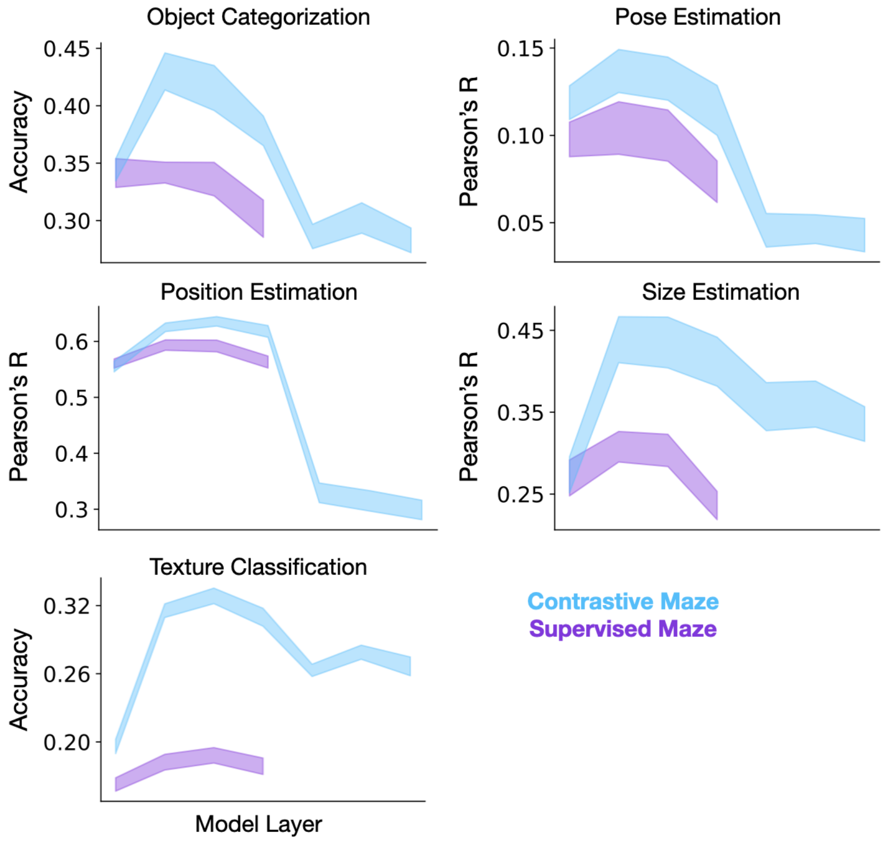

Fig 4 inset. Are those all supervised? (they are all black)
Yes, all those models are supervised on ImageNet categorization.

"We believe that future work could investigate other appropriate low-pass filters and ecologically-relevant pixel-level transformations to apply to the original image or video stream" - The authors may want to cite some existing work in this direction such as https://www.mdpi.com/2079-9292/10/22/2883 and https://www.biorxiv.org/content/10.1101/2020.06.16.154542v2
Thank you for the pointer! We have now cited both of those references in that sentence on pg. 15 of the revised manuscript at the quoted sentence, as suggested.

I assume the authors will be publishing their code upon publication of the paper (as per PlosCB policy) but right now it says it is only available via email.
Yes, we definitely plan to have a public repository once that paper is public that includes pretrained model checkpoints and analysis code. Additionally, at the time of initial submission, per the journal guidelines (https://journals.plos.org/ploscompbiol/s/code-availability), we shared a private version of our repository (https://github.com/neuroailab/mouse-vision) to the PLoS Comp editorial board email address (ploscompbiol@plos.org) to share with reviewers, but it does not seem this repository was forwarded to the reviewers. We will make this same repository link public upon acceptance, and have included this link in the "Code

Availability" section of the revised manuscript. In the meantime, we have also uploaded it as a zip file under "Supporting Information -- Compressed/ZIP File Archive".

Additional analyses:

Looking at the hierarchy score (e.g. https://www.sciencedirect.com/science/article/pii/S2589004221009810), based on the rough hierarchical categorization the authors find in S1B would be a helpful additional analysis that may reveal other trends in the impact of architecture and training. From what has already been plotted it seems possible that self-supervised learning better recapitulates the visual hierarchy as well.
You are correct. We performed a new analysis by evaluating the AlexNets, VGGs, and ResNets (of varying depths and objectives) using the brain hierarchy score you suggested. We find that the self-supervised, contrastive models (and shallower models) have a higher brain hierarchy score (computed using the mapping from model features to electrophysiological responses, i.e., encoding method; see figure below). In fact, the AlexNets (across all objective functions) perform the best on this metric.

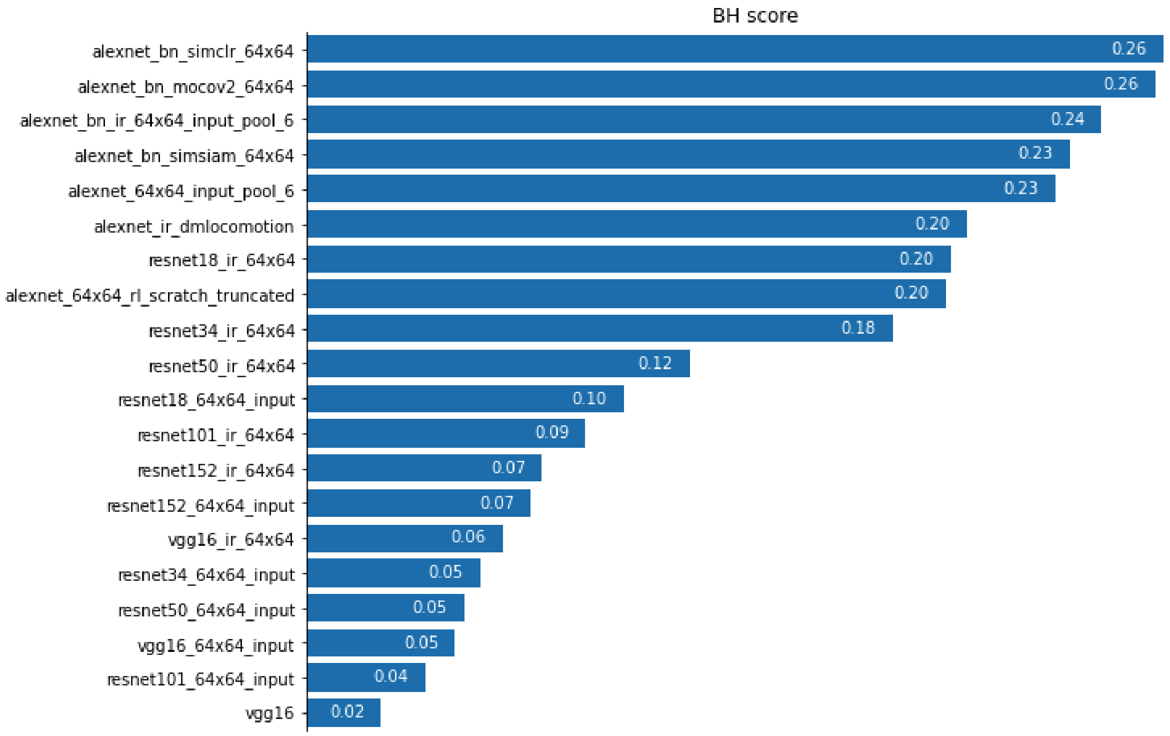

Why is there no self-supervised MouseNet? (also the plotting of MouseNet performance in Figure 1 has some other bar under it)
The MouseNet of Shi et al. (2022) was originally proposed to be trained in the supervised categorization context, which is why we trained it on ImageNet with this supervised objective. However, this is a great suggestion by the reviewer, and we have now trained MouseNet with the self-supervised objective (Instance Recognition), and found that

although this improves over its supervised counterpart, it does not outperform the neural predictivity of our original Contrastive AlexNet model (which was also trained with Instance Recognition) – which we note in the first paragraph of Section 3.1, when the MouseNet results are originally mentioned. We thank the reviewer for this suggestion, as it strengthens our results.

The second green bar in the new Figure 2A (previous Figure 1A) refers to our variant of MouseNet where everything is the same except that image categories are read off of at the penultimate "VISpor5" layer of the model (rather than the concatenation of the earlier layers as originally proposed). We thought this might improve the model's predictivity, since it can be difficult to train linear layers when the input dimensionality is very large. We apologize for the confusion, and have clarified this bar in footnote 2 on pg. 5 of the revised manuscript, when the MouseNet is first mentioned. We have also trained this version with Instance Recognition as well, and it also does not match the neural predictivity of our original Contrastive AlexNet model.

We have included the results for the two self-supervised MouseNets in the revised version of the manuscript, both as additional red bars in Figures 2A, S2, and S3; and as entries in Table S3.

Reviewer #3

Summary Of Contributions

The paper presents a computational model of the mouse visual cortex and finds that a shallow network with a low-resolution input is optimal for modeling mouse visual cortex. The authors find that models trained with self-supervised contrastive objectives are better matches to mouse cortex than models trained on supervised objectives or non-contrastive self-supervised methods. They show that the self-supervised, contrastive objective builds a general-purpose visual representation that transfers better to out-of-distribution visual scene understanding and reward-based navigation tasks.

Strengths And Weaknesses

Strengths

+ Paper is overall well organized
+ Convincing evidence presented for the main claims
+ Provides a nice comparative view on mouse vs. monkey visual system

Weaknesses

- There may be an issue with image size and resolution
- Writing and presentation of results can be improved to make the paper more accessible

Detailed Comments

The paper presents a thoroughly executed study whose main claims are well supported by the evidence presented. I am generally very supportive of the paper and have only two main concerns which I consider crucial to be addressed in a revision:

1. You make a claim about 64 px being the optimal image size for training. This sounds reasonable given the visual acuity and field of view of the mouse you cite in section 2.3. However, the argument cites a visual field of 60–90°, but the images spanned 120° in the experiments (according to the technical white paper on the dataset). Thus, the image is highly distorted, the resolution depends a lot on the eccentricity and I am not quite sure if the math actually works out. It would be great to look at this a bit more carefully.
Thank you for your careful investigation into the experiment from which the neural responses were collected. We want to first note that regardless of the assumptions surrounding visual acuity and the field of view, the data in Figure 2 (where neural predictivity is plotted as a function of image resolution at which the architecture was trained) show that an image size of roughly 64 px to 84 px results in the best neural predictivity for both the Contrastive AlexNet and the Dual StreamNet. For computational-tractability reasons, we unfortunately cannot perform such an analysis for all the models (i.e., we would have to train each model ~10 times for each of the ~10

image resolutions). Thus, we assume that training all models at 64 px is close to optimal for neural predictivity. This also allows us to be consistent with how models are trained and evaluated by Bakhtiari et al. (2021) and by Shi et al. (2022).

Regarding the visual field coverage, it is true that the image presentation spanned approximately 120 degrees. However, the receptive fields of each unit do not span that angle. This is based on data from Extended Data Fig. 6d and 6e in Siegle et al. (2021) describing the receptive fields of a single unit (figure shown below for convenience). From the figure, we can see that the estimated receptive fields do not span the entire visual field (panel e, middle column). Rather, the diameters of the receptive fields shown in the figure cover roughly 60-100 degrees. Thus, these data lend support to our assumption of a visual field coverage of less than ~64 degrees.

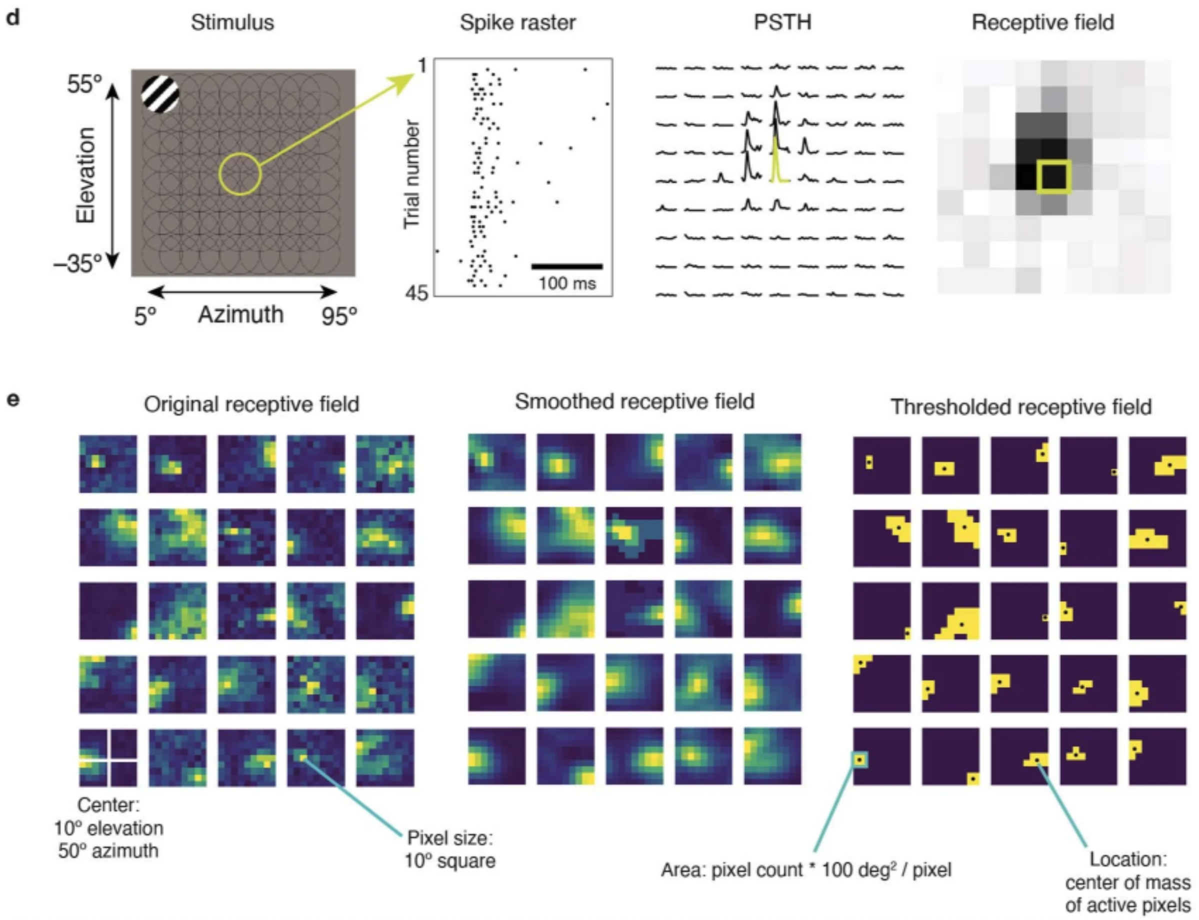

2. While the paper is overall very well organized, I found the results more difficult to read and digest than necessary and the methods sometimes incomplete. The following examples illustrate my issues, and I would highly encourage the authors to revise sections 2, 3 and 5 from the perspective of a reader who is not already familiar with the work and all the details.

- Section 2.1 is quite confusing. It's not clear to me what this section wants to accomplish. The claim "We identified the best-performing mapping function by assessing a variety of functions to map responses from one animal to those of another" is unclear to me. Why do you find the best-performing mapping function that way? Why would one want to map responses from one animal to another? If this is so central that you start discussing it as the first point after the intro, why don't you show any data supporting your claim (only something hidden in Fig. S1, but I couldn't figure out what exactly it shows and how it supports the claim).
We chose PLS regression to ascertain the level of neural response variance that is common across animals and that therefore should be "explained" by candidate models. This analysis helps us ensure that models are at least as similar to a given animal's neural responses as two conspecifics' neural responses are to each other. Therefore, we want to ensure we are studying the inter-animal similarity with high fidelity, to be in a position to best separate models. Furthermore, the PLS regression mapping confers the additional benefit of enabling direct comparison to prior primate ventral stream results (which also used this mapping; Yamins et al., 2014; Schrimpf et al., 2018) in order to better understand ecological differences between the two visual systems across mouse and primate species. We greatly expand on this motivation for the analysis in Section 2.1 as its own earlier Section 2, and move previous Figure S1 to the main text as Figure 1. We also include RSA results in the Appendix, and observe similar trends as with PLS regression which we note in the beginning of Section 3.1 in the revised manuscript.

- The text sometimes refers to the "prior primate model" (e.g. Section 2.2, first paragraph), but I don't think this concept has been clearly introduced. I can only guess "prior primate model" means VGG-16?
Yes, "prior primate model" refers to a supervised VGG16 model trained on 224 px inputs. We have clarified this in the figure captions and explicitly in the first paragraph of Section 3.2 on pg. 5 (including footnote 3), and thank the reviewer for the suggested clarification.

- The results of Fig. 1A could be presented in a more systematic way. Rather than just sorting by performance and some rough grouping, I think it would be better to systematically investigate individual factors while keeping all others constant. For instance, the best-performing is ShallowNet trained with IR. What happens you replace IR by other objectives? I found a few other contrastive objectives, but where is ImageNet-supervised ShallowNet? Is that the same as supervised AlexNet? If so, why do you call them differently? Similarly, keeping IR as the objective, how does architecture affect performance? I think in this case the relevant data points are shown in Fig. 1A, but it's quite tedious to find them.
We thank the reviewer for their suggestions here. We added a new supplemental figure (Figure S9) to make more clear how neural predictivity differs when either the architecture (left) or the objective function (right) is fixed. We also reference this figure in Sections 3.2 and 3.4. The figure is shown below for convenience. As can be seen below, AlexNet trained using instance recognition best predicts the mouse visual responses.

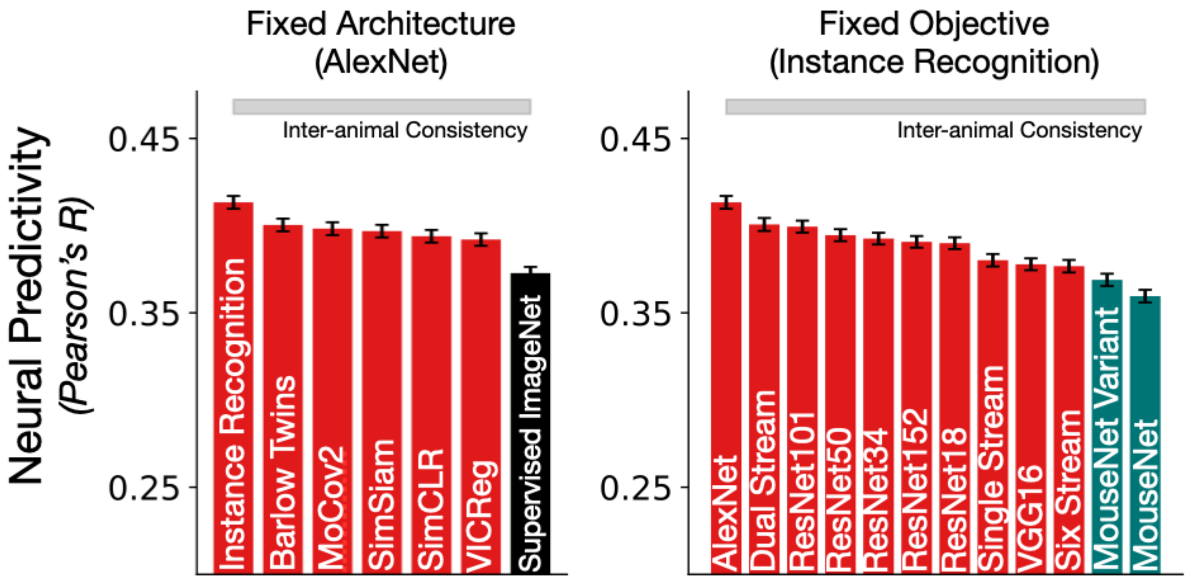

- The discussion of objectives in 2.2 is nice, but again I found it difficult to map to Fig. 1A. Categorization with 10 classes (Krizhevsky 2009) probably refers to CIFAR-10, depth prediction (Zhang 2017) probably to the orange bars, sparse autoencoding (Olshausen and Field 1996) probably pink. Why don't you state these things?
We apologize for the lack of clarity. We now make it more clear in Section 3.2 (previous Section 2.2) what colored bar (in Figure 2) each objective refers to. Thank you for this suggestion.

- What are the single-, two- and six-stream architectures and how do they relate to Olshausen and Field 1996 (which doesn't actually use a CNN or even an explicit encoder)?
We apologize for the confusion. The main relation to Olshausen and Field (1996) is the use of a sparsity penalty in the latent space of the image reconstruction loss. We now clarify this connection in Section 3.2 (previous Section 2.2) when this loss function is introduced. "Single Stream", "Dual Stream", and "Six Stream" are novel architectures we developed based on the first four layers of AlexNet, but additionally incorporates dense skip connections, known from the feedforward connectivity of the mouse connectome (Harris et al., 2016; Knox et al., 2018), as well as multiple parallel streams (schematized in Figure S1). We explicitly define this in the new Figure 2 caption (previous Figure 1), as well as in Section 3.3 when StreamNets are focused on.

- It's not quite clear to me whether "Contrastive ShallowNet" refers to just taking the first four layers of an entire AlexNet trained using IR or whether you take only the first four layers of AlexNet and add a global average pooling + fully connected layer in order to train IR. Please make the description a bit more explicit in Section 2.2 and the caption of Fig. 1.
Contrastive ShallowNet refers to the first four layers of an entire AlexNet trained using IR. Following your suggestion, we have reverted to calling it "Contrastive AlexNet" for clarity. We also developed novel four-layer architectures called the single-, two-, and six-stream StreamNets and trained them with different self-supervised objectives. We

have made the description more explicit both in the caption of new Figure 2 in the revision, and in the second paragraph of Section 3.3 of the revision. The StreamNets are also schematized in Figure S1.

- Related to previous point: I couldn't find a description in the methods how the StreamNet architecture (and ShallowNet) get from (3x3) feature maps to a single latent vector that is needed for, e.g., the contrastive losses or the classification objective. Global average pooling over space? Followed by a fully-connected layer? The sentence "Finally, the outputs of each parallel branch would be summed together, concatenated across the channels dimension, and used as input for the readout module" is very ambiguous? Summed across which dimension? What's the "readout module"?
We apologize for the lack of clarity and for not describing the readout module. We have now added the description of the module in Section 6.4 and clarified the ambiguous description of how features are obtained from the StreamNet architecture.

The readout module consists of an (adaptive) average pooling operation that upsamples the inputs to 6x6 feature maps. These feature maps are then flattened, so that each image only has a single feature vector. These feature vectors are then fed into a linear layer either for classification or for embedding them into a lower-dimensional space for the contrastive losses.

Prior to the readout module, the output of each (parallel) branch is a three-dimensional matrix (i.e., n_channels x height x width). The shape of each output is identical so that they can be summed together resulting in a single output matrix of dimensions (n_channels, height, width). For the StreamNets with two and six parallel streams (see Figure S1), there are two output modules (each associated with a single "Sum" operation). The outputs of those modules are concatenated across the channel dimension prior to the readout module.

- The StreamNet architecture is first mentioned in section 2.3, and was not at all clear to me from this section. I had to search for it in the methods to understand the architecture (somewhat). I'm still confused about what you mean by "StreamNet incorporates dense skip connections" - where are these skip connections? They are not mentioned in the methods section 5.4.
The skip connections of the StreamNet architecture describe the fact that the output of the first convolutional layer is convolved with a trainable filter and is summed with the output of the final convolutional layer. This is shown in Figure S1 (previous Figure S2).

- It didn't become clear to me why you can train AlexNet only on 64 px upwards by Contrastive StreamNet can be trained on 32 px. You could simply remove, e.g., the last max pooling layer.
If we remove the last max-pooling layer, the architecture is no longer an AlexNet, which is why the smallest image size we could use to train the AlexNet architecture is 64 px. Because of that, we used the StreamNet (which is not restricted by the final max-pooling

layer since it is based on the first four convolutional layers of AlexNet) to show that training on inputs that are *too* small lead to comparatively poor neural predictivity.

Other Minor Issues:

- Abstract: second sentence appears broken: "However, an overall understanding of the mouse's visual cortex, and how it supports a range [OF?] behaviors, remains unknown."
Thank you for pointing that out! We have fixed it in the abstract.

- The claim "We also attain neural predicitivity improvements over prior work...": Where is the data supporting this claim? The sentence references five papers, but I can only find MouseNet in Fig. 1. If some of the models in Fig. 1 map onto the references in this sentence, please state it explicitly somewhere so your readers can make the link.
The main data points supporting this claim are the MouseNet performance (as you mentioned in Figure 2 [previous Figure 1]) and also the CPC model of Bakhtiari et al. (2021). We now explicitly indicate that these are the purple and green bars in the revision.

- Fig. 1 caption: "All models are trained on 64 px inputs unless otherwise stated in figure." It's no where stated otherwise, only explicitly 64 px for VGG-16. Does this mean all models trained on 64 or did you forget to state it somewhere? CIFAR-10 has 32x32 images. Does that mean you're upsampling them to 64?
You are correct. All models are trained with 64 px inputs, except for the prior primate model, which is a supervised VGG-16 model trained with 224 px inputs. Yes, you are correct in that to train the models on CIFAR-10, the images are upsampled to 64 px for fair comparison.

- You repeatedly make the point that the categories of ImageNet are human-centric and not relevant for mice. But if that's the case, why would ImageNet be a good approach for monkeys, for whom ImageNet categories should be equally irrelevant?
We apologize for our confusing presentation of the motivation. The affordances of monkeys involves being able to finely manipulate objects flexibly with their hands (unlike mice). Therefore, being able to reliably recognize a large number of categories of objects (as encapsulated by the ecological pressure of 1000 ImageNet categories) may be more relevant to non-human primates as an ethological proxy than it is to mice. We clarify this as an additional footnote (4) to the sentence that mentions human-centricity on page 7. We thank the reviewer for getting us to clarify this point.
